# Microhardness Recovery and Micromorphology of Demineralized Dentin Restored with Modified Glass Hybrid Material

**DOI:** 10.3390/ma19091733

**Published:** 2026-04-24

**Authors:** Ivan Šalinović, Maja Bilić-Prcić, Maria Bota, Anja Ivica, Ivana Miletić

**Affiliations:** School of Dental Medicine, University of Zagreb, Gundulićeva 5, 10000 Zagreb, Croatia; isalinovic@sfzg.unizg.hr (I.Š.); mbota@sfzg.unizg.hr (M.B.); aivica@sfzg.unizg.hr (A.I.); miletic@sfzg.unizg.hr (I.M.)

**Keywords:** glass hybrids, bioactive glass, short glass fibers, dentin remineralization, microhardness

## Abstract

This study evaluated how the addition of 5 wt% bioactive glass and 15 wt% short glass fibers to EQUIA Forte HT affects the microhardness, micromorphology, and elemental composition of demineralized dentin. Class I cavities in 28 human third molars were demineralized with 37% phosphoric acid and restored with: (1) Filtek Universal composite, (2) EQUIA Forte HT, (3) EQUIA Forte HT + 5wt% BAG, or (4) EQUIA Forte HT + 15wt% short glass fibers. After 4 weeks of storage in phosphate-buffered saline at 37 °C, the teeth were cut in half, obtaining two samples from each tooth (*n* = 14). Vickers microhardness (HV0.1) was measured on demineralized dentin 50–100 μm apical to the restoration interface. Representative specimens (*n* = 2) were examined using scanning electron microscopy (SEM) and energy-dispersive X-ray spectroscopy (EDS). Data were analyzed with one-way ANOVA (α = 0.05). Unmodified EQUIA Forte HT showed the highest mean dentin microhardness recovery (25.06 ± 1.42 HV0.1), followed by composite (17.31 ± 0.66 HV0.1), BAG-modified (23.74 ± 1.37 HV0.1) and fiber-reinforced (22.15 ± 1.06 HV0.1) groups (*p* < 0.001, all pairwise comparisons *p* ≤ 0.039). Glass hybrids showed prominent Ca/P peaks; modified groups had elevated Si (BAG) and Al (fibers). SEM revealed smoother surfaces with fewer cracks in modified materials. Unmodified EQUIA Forte HT produced the highest short-term microhardness recovery, while BAG and fiber additions altered surface morphology and elemental composition but slightly reduced early hardness.

## 1. Introduction

Dental demineralization is a process characterized by the dissolution of inorganic apatite, widening of dentinal tubules, and gradual exposure of the collagen matrix and also serves as a key pathological event in dental caries development [1,2]. Acid-producing biofilms quickly undermine the structure of dentin, which consists of a collagen scaffold reinforced by nanoscale hydroxyapatite crystals. As mineral loss continues, the mechanical properties of dentin, including hardness, elastic modulus, and resistance to deformation, decline substantially [3,4]. The activation of endogenous proteases, such as cysteine cathepsins and matrix metalloproteinases (MMPs), which break down exposed collagen fibrils and reduce the possibility of tissue repair, further exacerbates structural weakening [5]. In contrast to enamel, where remineralization primarily involves surface mineral buildup, dentin repair depends on coordinated mineral deposition inside collagen fibrils and between them to regain its full mechanical strength [3,4]. This process is further challenged by fluctuating pH, limited ionic supersaturation and ongoing microbial activity, all of which hinder stable nucleation and growth of hydroxyapatite crystals [6,7,8]. Because many current remineralization strategies frequently restore only superficial mineral layers while deeper regions remain biomechanically unstable [3,4,5], demineralized dentin often fails to regain structural integrity.

Ion-releasing restorative materials have gained attention for their ability to deliver therapeutic ions, neutralize acidic environments and chemically bond to dentin [9]. However, many conventional ion-releasing materials lack either adequate bioactivity or sufficient mechanical reinforcement [9,10,11]. Conventional glass ionomer cements provide sustained fluoride release and chemical adhesion, but their brittleness, low fracture toughness and susceptibility to wear limit long-term clinical reliability [9,10,11,12].

To address these shortcomings, glass hybrid systems incorporating highly reactive glass particles and advanced polyacrylic acids have been developed to improve matrix crosslinking, strength and stability [12]. Fluoride-containing bioactive glass (BAG) is a particularly promising additive because it can release calcium, phosphate, fluoride and silica ions, elevate local pH and promote the formation of a silica-rich layer that subsequently converts into carbonated hydroxyapatite [13,14,15,16]. BAG incorporation has been shown to enhance microhardness, mineral density and ion exchange at the dentin–material interface [14,15], and to promote deeper and more stable remineralization [13,16]. Short glass fibers provide a complementary modification by increasing flexural strength, elastic modulus and crack-arresting capability [17,18]. Fiber reinforcement improves stress distribution, reduces catastrophic failure and enhances long-term mechanical performance [17,18]. When combined with bioactive fillers, fiber reinforcement may counterbalance any reduction in cohesive strength while contributing to enhanced mechanical and chemical stability [17]. These considerations highlight the need to evaluate the individual and combined effects of bioactive and mechanical enhancements within glass hybrids [19,20].

Microhardness analysis is often viewed as a highly reliable and non-destructive approach for assessing hard dental tissues mineralization processes, widely used in previous studies [21,22]. Further surface morphology data, as well as elemental analysis, are commonly obtained by scanning electron microscopy (SEM) and energy-dispersive X-ray spectroscopy (EDS) [23,24].

Therefore, the aim of this study was to assess the effects of incorporating 5 wt% bioactive glass and 15 wt% short glass fibers into a commercial glass hybrid restorative material on the microhardness, micromorphology and elemental composition of demineralized dentin. The null hypotheses were:

1. There is no difference in dentin microhardness among the unmodified glass hybrid, bioactive glass-modified, fiber-reinforced and composite control groups.
2. There are no differences in elemental composition, including Ca, P, F and Si, among the four restorative groups.
3. There are no differences in micro surface morphology among the groups as assessed by scanning electron microscopy (SEM).

## 2. Materials and Methods

### 2.1. Sample Preparation

Twenty eight sound human third molars extracted for orthodontic or surgical reasons were collected following informed consent, inspected under 2.5× magnification using a surgical loupe (Carl Zeiss Meditec AG, Jena, Germany) to exclude cracks, restorations or structural defects, and stored in 0.5% chloramine solution (Kemika d.d., Zagreb, Croatia) at 4 °C for up to three months. Before cavity preparation, teeth were cleaned with rotating brushes (KerrHawe, Bioggio, Switzerland), a hand scaler (Hu-Friedy Mfg. Co., Chicago, IL, USA) and abrasive disks (Sof-Lex, 3M ESPE, St. Paul, MN, USA) to remove debris. Occlusal enamel was removed using a sectioning machine IsoMet 1000 (Buehler, Lake Bluff, IL, USA) paired with a diamond-coated cutting blade. The rotating speed was set at 250 per minute. Standardized Class I cavities (3 × 3 × 2 mm) were prepared in mid-coronal dentin using a medium-grit cylindrical diamond bur (Komet 835KR, Komet Dental, Lemgo, Germany) mounted in a water-cooled high-speed turbine, with a new bur used after every five preparations to maintain consistent cutting efficiency. Half of each cavity was covered with acid-resistant nail polish (Markwins Beauty Brands, Inc., Walnut, CA, USA) to allow intra-tooth comparison of treated and untreated surfaces, as previously described. Demineralization was performed using 37% phosphoric acid gel (Ultradent Products Inc., South Jordan, UT, USA), applied in three consecutive 60 s cycles with thorough rinsing and gentle air-drying between applications. Samples for four experimental restorative groups were prepared (*n* = 14 per group):

1. Filtek Universal composite (3M ESPE, St. Paul, MN, USA) as the non-ion-releasing control, containing aromatic urethane dimethacrylate (AUDMA), additive fragmentation monomer (AFM), diurethane dimethacrylate (DMA), and 1,12-dodecane-DMA as the resin matrix. The inorganic filler system comprises a combination of non-agglomerated/non-aggregated 20 nm silica filler, non-agglomerated/non-aggregated 4–11 nm zirconia filler, aggregated zirconia/silica cluster filler (consisting of 20 nm silica and 4–11 nm zirconia particles), and agglomerated ytterbium trifluoride particles (~100 nm), with a total inorganic filler loading of approximately 76.5 wt% (58.4 vol%).
2. EQUIA Forte HT (GC Corporation, Tokyo, Japan) as control, containing: fluoroaluminosilicate glass, polyacrylic acid, iron oxide (powder); polybasic carboxylic acid, water (liquid).
3. EQUIA Forte HT supplemented with 5 wt% 45S5 commercial bioactive glass (Schott AG, Mainz, Germany), particle size of 4.0 μm.
4. EQUIA Forte HT supplemented with 15 wt% short glass fibers (Central Glass Fiber Co., Ltd., Tokyo, Japan), 6 µm diameter, average length of 140 µm.

The sample size (*n* = 14 specimens per group) was selected based on the dentin microhardness literature using 10–15 specimens and confirmed by a priori power analysis (G*Power; f = 0.25, significance level 0.05, power = 0.80), requiring 13 specimens per group for one-way ANOVA with four groups. For the modified materials, bioactive glass or short glass fibers were homogeneously incorporated into the powder component of the glass hybrid material prior to mixing with the liquid, and each material was applied according to the manufacturer’s instructions. Following placement, all specimens were stored in phosphate-buffered saline (PBS, pH 7.4) at 37 °C in an incubator ES 120 (NÜVE, Ankara, Turkey) for four weeks to allow ion exchange between the restorative material and dentin. Each specimen was then embedded in silicone molds filled with cold-cure acrylic resin (Heraus Kuzler GmbH, Hanau, Germany) to provide stability and ensure precise orientation during sectioning. After polymerization, the embedded teeth were sectioned longitudinally through the center of the restoration using a low-speed, water-cooled diamond precision saw, minimizing heat generation and preventing structural alterations of dentin. Thus, from each tooth, two samples were obtained. The principle of obtaining multiple samples from a single tooth was adapted from previous studies [25,26,27].

### 2.2. Microhardness Testing

Dentin microhardness was determined using a Vickers microhardness tester KBW 1-V (KB Prueftechnik GmbH, Hochdorf-Assenheim, Germany), with a Vickers indenter applying a 100 g load (HV0.1) for 10 s. Three indentations were made on each specimen, spaced at least three indentation diameters apart and located 50–100 μm apical to the dentin–material interface. Mean values were used for statistical analysis.

### 2.3. SEM and EDS Analysis

SEM-EDS was used to provide both structural and elemental analysis, enabling direct quantification of calcium and phosphate ratios and detection of ion exchange, which are confirmed markers of dentin remineralization in earlier studies [23,28]. Representative specimens (*n* = 2 per group) were gently polished using a nylon brush (KerrHawe, Bioggio, Switzerland), air-dried, mounted on aluminum stubs (Agar Scientific Ltd., Essex, UK) and sputter coated with gold using a Q150R S sputter coater (Quorum Technologies Ltd., Lewes, UK). The images of the surface were obtained using the microscope model JSM-7000F (JEOL Ltd., Tokyo, Japan), with the accelerating voltage of 5 kV and working distance of 10 mm. EDS was performed using the Inca 350 EDS system (Oxford Instruments, High Wycombe, UK).

### 2.4. Statistical Analysis

Statistical analysis was performed using IBM SPSS Statistics, version 20 (IBM Corp., Armonk, NY, USA). Data normality was assessed with the Kolmogorov–Smirnov test (*p* > 0.05). Intergroup differences were analyzed using one-way ANOVA, followed by the Scheffe post hoc test, with the level of statistical significance set at *p* < 0.05.

## 3. Results

### 3.1. Microhardness Measurement

Dentin microhardness (HV0.1) was measured on demineralized dentin surfaces, both with and without acid-resistant varnish coverage. For the varnished (exposed) demineralized surfaces, intergroup differences were not statistically significant (ANOVA, *p* = 0.439), and no significant correlation was detected between varnished and unvarnished measurements. Consequently, subsequent material comparisons were performed on the unvarnished measurements. Obtained mean microhardness values are shown in Table 1.

Differences among the groups were statistically significant overall (ANOVA, *p* < 0.001), and between all pairwise group comparisons (*p* = 0.039 for the difference between EQUIA Forte HT and EQUIA Forte HT + 5 wt% BAG, *p* < 0.001 for all other pairwise comparisons). EQUIA Forte HT showed the highest microhardness, followed by EQUIA Forte HT + 5 wt% BAG and EQUIA Forte HT + 15 wt% glass fibers. The composite control exhibited significantly lower microhardness at the interface.

### 3.2. EDS Analysis

**Figure 1:**
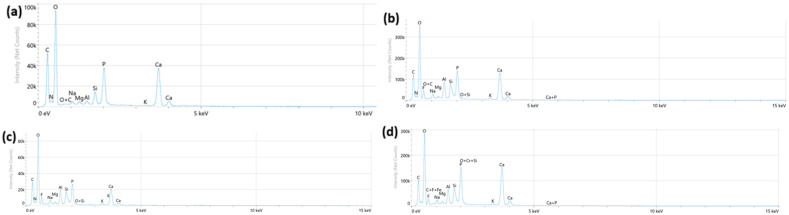
Representative EDS spectra from the dentin surface adjacent to the restorative material; (**a**) composite control; (**b**) EQUIA Forte HT; (**c**) EQUIA Forte HT + 5 wt% BAG; (**d**) EQUIA Forte HT + 15 wt% fibers.

Different elemental signatures consistent with ion exchange and remineralization mechanisms were found in EDS spectra obtained at the dentin–material interface (Figure 1a–d). Significantly higher Si in both modified groups indicated bioactive glass dissolution, while all glass hybrid groups showed prominent Ca and P peaks suggestive of ion accumulation, but do not confirm crystalline formation. The fiber-reinforced group was dominated by Al. The composite control displayed baseline apatite peaks.

### 3.3. SEM Analysis

**Figure 2:**
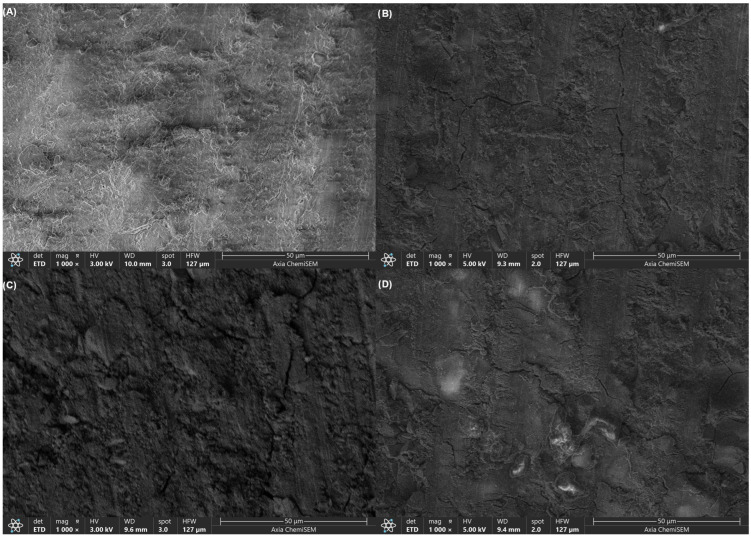
SEM images (1000×) of demineralized dentin surfaces at the material–dentin interface after 4 weeks. (**A**) Composite control, (**B**) EQUIA Forte HT (unmodified), (**C**) EQUIA Forte HT + 5 wt% bioactive glass, (**D**) EQUIA Forte HT + 15 wt% short glass fibers.

Images of the dentine surface obtained with scanning electron microscope show clear differences among the experimental groups. In the composite control group, irregular topography with extensive white layer coverage of varying shapes and sizes and prominent linear cracks was found on dentine surface (Figure 2A). Unmodified EQUIA Forte HT restoration produced dentine surfaces with scattered white patches of irregular dimensions and minor linear cracks running parallel to the surface features, with relatively uniform surface morphology (Figure 2B). EQUIA Forte HT modified with 5 wt% bioactive glass resulted in surfaces with more continuous white coverage extending across broader regions, substantially reduced prevalence of linear cracks, and somewhat rougher texture compared to both control groups (Figure 2C). The fiber-reinforced formulation (EQUIA Forte HT + 15 wt% short glass fibers) showed scattered white patches of irregular dimensions and only isolated cracks (Figure 2D).

## 4. Discussion

The results of this study suggest that modification of the glass hybrid restorative with either BAG or short glass fibers significantly changes its interaction with demineralized dentin, as reflected in changes in surface microhardness, micromorphology and elemental composition at the material–dentin interface. Consequently, all null hypotheses stating that these modifications would have no effect on microhardness, elemental distribution or surface morphology were rejected.

In the present experiment, dentin restored with unmodified EQUIA Forte HT showed the highest mean microhardness values (25.06 ± 1.42 HV0.1), which were statistically significantly higher than those measured for the composite control (17.31 ± 0.66 HV0.1), BAG-modified (23.74 ± 1.37 HV0.1), and fiber-reinforced (22.15 ± 1.06 HV0.1) groups (ANOVA, *p* < 0.001). Pairwise comparisons confirmed significant differences between all groups (*p* = 0.039 for EQUIA Forte HT vs. BAG-modified; *p* < 0.001 for all other pairs), with EQUIA Forte HT outperforming even the modifications. These microhardness results align with previous findings where EQUIA Forte HT demonstrated superior remineralization potential compared to other glass ionomers and alkasites, achieving values up to 37.74 HV0.1 after 28 days [29]. Both groups with modifications lead to lower microhardness increase compared to the unmodified glass hybrid group, though still exceeding the composite control. This outcome was expected, as Filtek Universal is a resin-based composite that does not release ions and therefore cannot contribute to dentin remineralization, unlike glass hybrid materials whose ion exchange capacity is the primary driver of microhardness recovery in demineralized dentin. Therefore, under the study conditions, BAG and fiber did not improve early microhardness recovery, even though they changed interfacial chemistry and micromorphology.

SEM observations at 1000× confirmed these mechanical findings, revealing progressive changes in surface homogeneity from the cracked, irregular control surfaces to the smoothest fiber-reinforced morphology. EDS analysis corroborated this trend, with spectra showing baseline Ca/P peaks in the control but elevated Si and Al specifically in the modified glass hybrid groups. Fluoride peaks were consistent across all glass hybrid materials. These results suggest that, while the unmodified glass hybrid matrix provides optimal hardness recovery, BAG and fiber incorporation can possibly offer compositional and mechanical improvements, further boosting the effect of the material [30]. However, SEM/EDS analysis cannon fully determine crystalline structure or subsurface mineralization, as noted in the limitations.

The superior microhardness recovery seen with unmodified EQUIA Forte HT over the composite control most likely comes from its distinctive ion exchange process, where polyacrylate chains in the glass hybrid matrix enable Ca^2+^, PO_4_^3−^, and F^−^ ions to diffuse into demineralized dentin [31]. This forms an ion exchange layer at the interface that drives apatite formation, as confirmed by the fluoride peaks in EDS spectra from all glass hybrid groups. The modest hardness drop with BAG (23.74 HV0.1) or fiber (22.15 HV0.1) addition likely arises from diluting the primary reactive glass powder, the key source of ongoing ion release in the unmodified material. With fibers mostly confined to the surface in the set cement, they may also restrict ion pathways, weakening the concentration gradient for ion exchange [32]. The observed difference in microhardness recovery is a common trade-off in ion-releasing restorative materials; enhancing bioactivity or mechanical reinforcement often comes at the expense of early ion exchange efficacy [33]. The incorporation of BAG usually enhances surface bioactivity by promoting a rapid ion release and silica gel formation, which may improve long-term remineralization and pH buffering under acidic conditions. However, it also dilutes glass hybrid’s primary reactive phase, which can compromise short-term hardness recovery [33]. On the other hand, fiber reinforcement improves flexural strength and crack resistance—critical for load-bearing restorations—yet may impede ion diffusion due to surface confinement and altered porosity [34]. Clinically, unmodified EQUIA Forte HT may be preferable for immediate dentin stabilization in small cavities, while modified versions could benefit high-risk patients needing sustained bioactivity or enhanced durability. Therefore, unmodified EQUIA Forte HT’s results are likely caused by its unobstructed ion delivery, which aligns with studies showing prolonged calcium, phosphate and fluoride release compared to modified formulations at neutral pH [35]. BAG dissolves in a specific way that complements the glass hybrid matrix in these materials [36]. When BAG particles get wet, they rapidly form a silica-rich gel layer on their surface. This layer attracts calcium and phosphate ions from the surrounding solution, which leads to the formation of carbonated hydroxyapatite (cHAP), a mineral similar to natural tooth structure, within the first 24 to 48 h [36]. The stronger silicon signals we saw in the EDS spectra from the BAG and fiber-reinforced groups confirm this process. The silicic acid (H_4_SiO_4_) released during dissolution acts as a scaffold that guides mineral growth, resulting in the smooth, even white deposits visible across larger areas in our SEM images [36,37].

That said, BAG’s fast initial breakdown can quickly use up nearby ions, unlike the more gradual, controlled release from the EQUIA Forte HT matrix itself. This difference probably explains why the BAG-modified samples showed slightly lower microhardness values, even though their surfaces looked smoother overall [38]. Other studies support this observation. They note that glass ionomer cements (GICs) modified with BAG typically reach hardness levels similar to standard GICs only after extended aging, often more than 90 days, once the silica gel network fully develops and stabilizes the cHAP layer [39]. This is supported by Heshmat et al., who reported that BAG-modified GICs are very effective in promoting dentin remineralization, but show lower hardness values in the short term due to this early ion burst effect [40].

Ion release from these materials correlates directly with the observed gains in microhardness, mainly through mineral deposition that fills microscopic gaps and overall densification of the matrix [14]. Fluoride from the glass hybrid component promotes faster hydroxyapatite (HAP) crystal formation by substituting for hydroxide ions, while calcium and phosphate ions work to restore the natural Ca/P ratio in dentin that was altered by phosphoric acid demineralization [41,42]. Silicon contributes more than just serving as a template for mineral growth. The silicate species released from its dissolution can bind to MMPs—enzymes that degrade collagen—and help stabilize the exposed collagen fibrils remaining after demineralization. This binding prevents further breakdown by these enzymes and preserves the structural framework needed for new mineral deposition [43]. Notably, even with only trace silicon detected in the unmodified EQUIA Forte HT, the glass matrix in this material still supplies small amounts that support early mineral nucleation, without triggering the more rapid, extensive dissolution seen with added BAG [44]. This gradual silicon release likely contributes to better hardness outcomes by limiting excessive gel formation, which might otherwise block pathways for other ions to reach the dentin surface [36].

Surface morphology differences, such as the distinct texture observed in the fiber-reinforced group, most probably arise from the mechanical reinforcement provided by the short glass fibers. These fibers distribute polymerization stresses more evenly during the setting process, which minimizes shrinkage-related microcracks that could otherwise propagate to the dentin interface [32]. Their aluminum and silicon composition can potentially also release ions following partial dissolution to increase local supersaturation for mineral growth [34].

This study had several limitations that should be acknowledged. The 4-week immersion in PBS at pH 7.4 and 37 °C simulates basic ion exchange under static conditions, but it does not capture the more complex dynamics of the oral environment, including saliva dilution, fluctuating pH levels, or mechanical forces from occlusal loading. Although SEM-EDS offered valuable information on surface morphology and elemental makeup, it does not evaluate subsurface ion distribution, mineral deposition within collagen fibrils, the mechanical properties of the hybrid layer or the crystalline nature of deposits. Nevertheless, the observed elemental and morphological changes, together with microhardness recovery results, provide indirect evidence of ion exchange and possible surface mineral deposition. We examined only specific concentrations of BAG (5 wt%) and fibers (15 wt%) at a single time point, which restricted our capacity to investigate dose–response effects or outcomes over extended periods. Thus, any potential advantages of BAG and fiber modifications may be more obvious in long-term studies, rather than in immediate microhardness recovery assessment. Additionally, in this study, two samples were obtained from one tooth. Such study designs reduce the number of teeth included, yet they are widely used when assessing dentin mechanical properties to reduce inter-tooth variability and maximize obtained information from each donor tooth [45]. Finally, the controlled lab setting presumed ideal handling and moisture management, overlooking the variations typical in clinical practice. Future studies could address these gaps by including multi-timepoint fatigue tests, pH-cycling protocols, and intraoral appliance models to enhance clinical applicability.

## 5. Conclusions

Under the conditions of this study, the unmodified EQUIA Forte HT showed the best dentin microhardness recovery potential overall. Adding BAG and fibers did most likely enrich the material composition; however, these modifications resulted in lower short-term microhardness values. This study provides an important quantitative assessment of how BAG and fiber additions together influence the glass hybrid–dentin interface after incubation.

## Figures and Tables

**Table 1:** Obtained dentine microhardness values, HV0.1 [-].

Control (Composite)		EQUIA Forte HT	EQUIA Forte HT + 5% BAG	EQUIA Forte HT + 15% GF
Exposed	17.31 ± 0.66	25.06 ± 1.42	23.74 ± 1.37	22.15 ± 1.06
Varnished	56.83 ± 1.97	56.31 ± 1.61	56.15 ± 1.74	55.71 ± 1.90

Hardness is a dimensionless parameter and reported without units.

## Data Availability

The raw data supporting the conclusions of this article will be made available by the authors on request.

## Abbreviations

MMP—matrix metalloproteinases; BAG—bioactive glass; PBS—phosphate-buffered saline; HAP—hydroxyapatite; SEM—scanning electron microscopy; EDS—energy-dispersive X-ray spectroscopy; AUDMA—aromatic urethane dimethacrylate; AFM—additive fragmentation monomer; DMA—diurethane dimethacrylate; ANOVA—analysis of variance; HV—Vickers hardness.
